# Care Needs and Care Options for Frail Older People Living Alone in Italy: An Exploratory Mixed Study

**DOI:** 10.3390/healthcare14111432

**Published:** 2026-05-22

**Authors:** Maria Gabriella Melchiorre, Marco Socci, Giovanni Lamura, Sabrina Quattrini

**Affiliations:** Centre for Socio-Economic Research on Ageing, IRCCS INRCA—National Institute of Health and Science on Ageing, Via Santa Margherita 5, 60124 Ancona, Italy; g.melchiorre@inrca.it (M.G.M.); g.lamura@inrca.it (G.L.); s.quattrini@inrca.it (S.Q.)

**Keywords:** ageing in place, frail older people, functional limitations, daily living activities, health, well-being, family, friends and neighbours, self-help/care, Italy

## Abstract

**Highlights:**

**What are the main findings of this study?**

- Due to ageing and functional limitations, older people living alone have several needs (e.g., regarding activities of daily living, health, and mobility).
- Most support is provided by relatives, mainly children. Additional support from friends and neighbours, home services, aids, and even self-care is also reported.

**What are the implications of the main findings?**

- This descriptive study highlights that available care options, mostly when they are combined/integrated, have a key role for older people’s well-being.
- Effective interventions can allow ageing in place and promote the well-being of seniors, especially when both the elderly care recipients and their family caregivers are supported.

**Abstract:**

**Background/Objectives**: People aged 65 years and older who live alone and have limited functional abilities need support in many circumstances and for a variety of activities. This study was conducted to explore the available formal and informal help for seniors, using findings from the “Inclusive Ageing in Place” (IN-AGE) study. **Methods**: This descriptive study was carried out in 2019 in three Italian regions, i.e., Lombardy in the north, Marche in the centre, and Calabria in the south, and involved 120 older people who lived at home, either alone or with a personal/private care assistant (PCA). Using a mixed-methods approach revealed both qualitative (thematic/content analysis of narratives) and quantitative (quantifications of statements) results. **Results**: This study identified several needs of seniors in different circumstances concerning basic and instrumental activities of daily living (ADL and IADL), health, and mobility in/outside the home. The seniors reported that support was provided primarily by their families, followed by friends and neighbours. Public home services were considered insufficient. The participants also reported using mobility aids and instances of self-sufficiency. **Conclusions**: These results highlight the need to improve support services for frail seniors and to better integrate formal and informal caregiving to facilitate ageing in place and promote the well-being of older people. Adequate interventions should be implemented for both older people and their family caregivers, who play a central role in care.

## 1. Introduction

Living alone at home, without cohabiting relatives, can be a difficult challenge for frail older people with functional limitations that negatively impact the ability to perform daily activities, with negative consequences for health outcomes [1]. Frailty spans several domains: socio-demographic status, economic status [2], health and multimorbidity [3], and available social support [4]. Despite these factors, ageing in place, i.e., “the ability to remain at home throughout late life” [5] (p. 1), can provide seniors with social connectedness and integration [6], in addition to delayed institutionalisation and improved well-being [7]. Approximately 51% of females and 24% of males aged 65 years and over live alone in a large portion of Europe, with Italy in almost complete alignment with these statistics (at 50% and 20%, respectively). Some authors have reported that seniors living alone are at high risk for poverty [8,9].

Seniors living alone need care and assistance. This situation will become more complicated in the future, in light of the increasing share of older people worldwide who are often affected by multimorbidity. The share of people aged 65 years and over is expected to reach 29% by 2050 (up from the current 22%) in the European Union (EU-27), with higher values in Greece, Italy, and Portugal (increasing from the current 24–25% to approximately 34–35%). Multimorbidity affects more than 40% of seniors in the EU-27 [10] and 52% in Italy [11] and typically increases with age [12].

As they age, people’s ability to manage their health and take care of their own well-being tends to decrease. Conversely, their need for health services, and help using them, including reading and understanding complicated medical instructions, increases. Primarily, support is needed for accessing healthcare services, including general practitioners (GPs), for several reasons, e.g., cost, waiting times, and distance to the closest health facility, resulting in a need for support and accompaniment [10,13,14]. In addition, possible health emergencies, e.g., heart/circulatory/respiratory problems and physical accidents, especially falls, can occur. Falls comprise around 70% [15] and 77% [16] of all injuries involving seniors in Europe and Italy, respectively, and happen mainly at home, potentially resulting in hip fractures [17,18]. Poor health can, in turn, reduce the ability to perform personal and domestic care [19], i.e., basic (e.g., dressing, bathing, eating) and instrumental (e.g., cleaning, taking medication, managing finances) activities of daily living (ADL and IADL) [20], with a consequent need for help in this respect. Recent data for 2024 from EUROSTAT [21] indicate that 47% of seniors aged over 65 years in the EU-27, and 37% in Italy, have long-standing limitations in performing daily activities due to health problems. Another important issue linked to the above is mobility, both inside and outside the home (in the neighbourhood) [22]. In particular, seniors’ mobility problems affect leaving the house, accessing buildings and the external built environment, and using public transport. As a result, mobility aids, such as walking sticks, crutches, walkers, and wheelchairs, have become very important [11].

Available resources and care arrangements/options are critical. Regarding resources, public spending on long-term care (LTC) is greater in northern Europe (e.g., Norway and Sweden) and western Europe (e.g., Belgium and the Netherlands), and lower in central (e.g., Poland and Bulgaria) and southern (e.g., Italy and Greece) Europe [23]. In Italy, 74% of total public spending on LTC is devoted to persons aged over 65. Of this percentage, only 19% concerns home care service (e.g., Servizio di Assistenza Domiciliare in Italian—SAD), whereas 51% is allocated to cash benefits (e.g., National Disability Attendance Allowance, Indennità di accompagnamento in Italian, IA) and 30% to beneficiaries residing in facilities [24]. These assets impact both provision and access to formal care services and, consequently, available care options, which differ among European countries. In the north, formal/institutional care is predominant, while informal/family care is predominant in the south [25]. In particular, caregivers in Europe with lower socioeconomic resources (e.g., education and income) are more likely to informally care for their older relatives; these circumstances are less frequent when social spending is greater [26].

Informal carers contribute about 80% of the assistance provided to older relatives across the EU-27 [23]. Italy is a southern European country in which families traditionally care for older members. Recently, in the period 2023–2024 [27], 95% of people aged over 65 with disabilities/frailties reported receiving help with daily activities from family members, 37% from a personal/private care assistant (PCA), and 12% from friends/neighbours/acquaintances. Additionally, 12% receive help from operators of professional home care (PHC) services, and only 2% receive assistance at a day centre. A small percentage are supported by volunteer associations (2%). With regard to Italian seniors with serious difficulties in personal care, 84% receive support from family members (cohabiting or not), i.e., 52% are cared for by only relatives, and 32% receive care from both relatives and PCAs and/or PHC operators and private services, e.g., domestic home help (DHH) [11,28]. However, the possibility of having paid assistance (e.g., PCA or DHH) is conditioned by one’s own financial availability, and it is more common among seniors with higher incomes. Cost also impacts the possibility of living in residential facilities in Italy, with only 2% of seniors residing in these care homes in 2023 [29]. Notably, in Italy, approximately 19% of seniors aged over 75 years and 15% of those living alone or with older partners report not receiving adequate assistance with their daily needs [30]. Therefore, some cases of self-sufficiency are also reported in the literature [31,32], with seniors trying to meet some of their own needs, despite functional limitations. This especially likely when relatives live far away, have work commitments, or when other forms of support, even paid/private, are not available. Some authors have stressed that self-care among older people is a key strategy for strengthening their functional abilities and even alleviating their feelings of loneliness [33].

As described above, the existing literature contains several studies on care arrangements for frail older people ageing in place, especially with respect to their health needs, ADL/IADL, and mobility. To our knowledge, a comparison of several possible needs, including for specific circumstances, e.g., health emergencies, is lacking, as is a comparison of several possible sources of help, including self-help and mobility aids. Therefore, the aim of our study was to address this knowledge gap regarding older people with limited physical functionality living alone in Italy. Findings from the “Inclusive Ageing in Place” (IN-AGE) research project [28] were examined to answer the following research questions: (1) What are the main difficulties of older people regarding, e.g., daily living activities, health, mobility, housing-related problems, and hiring a PCA? (2) What are the main supports available in this respect, e.g., relatives, friends and neighbours, operators from home services, PCA, and volunteers? It is presumed that, for the most part, the families are still the main pillar of support for these seniors, even though a mix of other types of supports may be used when help from the family is not available or not sufficient. The examination of available care options in different situations can provide useful insights for policymakers regarding their impact on older people’s health and well-being, thus aiding in the development adequate care interventions and prevention strategies to enhance seniors’ ability to live independently.

## 2. Materials and Methods

### 2.1. Settings

The qualitative IN-AGE survey was carried out from May to December 2019 at both urban and rural/inner sites in three Italian regions (Lombardy in the north, Marche in the centre, and Calabria in the south). It involved 120 older people, with a split of 40 respondents in each region, of which 24 and 16 respondents in urban and rural areas, respectively (Figure S1 in the Supplementary Material, File S1). Some peripheral urban districts with a high proportion of older people living in public housing [34], in addition to inner areas with increasing ageing populations [35], were included. Overall, the three regions selected as settings for the survey are representative of three diverse levels of socio-economic development in Italy: high in the north, medium in the centre, and low in the south [36].

### 2.2. Framework of the Study and Ethical Issues

A qualitative exploratory research approach was adopted for a better understanding of the behaviours and experiences of the elderly participants [37].

The framework and phases of the study (Figure S2 in the Supplementary Material, File S1). were approved by the Ethics Committee of the Polytechnic of Milan (POLIMI, Research Service, Educational Innovation Support Services Area), authorisation n. 5/2019, on 14 March 2019. The protocol was revised to address with ethical concerns raised by the European General Data Protection Regulation (GDPR) n. 679 of 27 April 2016 [38].

The senior participants were informed of the aim and ethical procedure of the study by means of an invitation letter, and they signed a written informed consent form agreeing to be interviewed. They were informed of the steps taken to protect their privacy and the anonymity of personal/sensitive information (alpha-numeric codes were used to represent each participant).

#### 2.2.1. Sample and Recruitment

A purposive (non-probabilistic) typological sample was established. There was no intent to ensure statistical representativeness, but the sample was established so that the characteristics of the participants permitted adequate exploration of the phenomenon [39]. The inclusion criteria identified frail seniors as follows: individuals of either gender aged 65 years and over; living alone at home (with no cohabiting relatives) or with the support of a PCA; with an intermediate level of mobility, ranging between being limited in the home and being outside with help (from persons or aids); without cognitive impairment (to be able to participate in the interview autonomously); and without help from close family members (i.e., living in the same urban block or rural building) [28].

The recruitment of participants was supported by local branches of Italian volunteer associations (Auser, Anteas, and Caritas) and public home care (social services) providers, who first verified the eligibility of potential older participants, particularly regarding their cognitive status and physical autonomy, assessed in accordance with their usual procedures. They also preliminarily provided seniors (and their respective families) with the necessary information about the study. The contact details (name, address, and telephone number) of those who expressed a willingness to participate were provided to the research team, who conducted the interviews with those who definitively agreed to participate. The study reached a participation rate of 58%. The reasons for seniors declining the interview, despite their initial availability, were illness or hospitalisation during the survey and reconsidering the interview. With regard to data saturation, recruitment was closed when no additional categories or codes were generated and, in turn, no relevant new information was gathered by reading the 120 transcripts.

#### 2.2.2. Instruments for Data Collection

Both a few close/quantitative questions (on socio-demographic characteristics, physical/functional limitations in daily living activities, and extension of the support network) and more in-depth qualitative questions (on available support/care options for different needs and circumstances) were included in the semi-structured interviews. These were based on items/questions adapted from previous studies and research instruments [40] in order to establish a preliminary conceptual framework derived from the literature.

Physical/functional limitations were assessed according to 12 activities of daily living, both ADL and IADL [20], integrated with two mobility limitations (going up/down the stairs and bending to pick up an object from the ground) and two sensory limitations (problems for hearing and eyesight) [11,41]. The participants were asked to indicate whether they performed each activity mentioned above autonomously/alone or with help (from persons/aids) or if they were incapable of the activity. The extension of their support network was determined as the total number of persons providing help (relatives, friends and neighbours, operators from home services, PCAs, and volunteers). In addition, the following open questions were asked: “Please, tell me your needs in some different circumstances, e.g., daily activities, health, emergencies with health consequences (e.g., falls/fractures), mobility, repairs in the home, and recruitment of a PCA”. “Please, can you tell me more about who (persons), and/or what (aids) supported you most in such circumstances?”. With regard to health, the following questions were asked: “Who is the main reference person for managing health needs”. “Who provided the first help in emergency circumstances?”. All needs (at least one episode and at least one help) were considered for the year preceding the interview except for health emergencies, which were considered for the three preceding years. The research team decided to record episodes that occurred up to three years before the interview to collect retrospective data, since some health emergencies (e.g., falls) can have lasting consequences [42].

The interviews were administered face-to-face by six researchers (two interviewers in each region; three psychologists and three sociologists; five females and one male) with deep expertise in qualitative surveys involving older people. Each interview lasted approximately 60–90 min, was conducted in the seniors’ homes, and was audio-recorded and transcribed in full/*verbatim* by the interviewers. The *verbatim* transcripts were not returned to the frail seniors participating in the study for review and appropriate validation to avoid causing unnecessary stress.

#### 2.2.3. Mixed-Methods Data Analysis

A mixed-methods data analysis, which was mainly descriptive, was carried out on the 120 interviews. There was no comparison among regions/sites, since the focus of the study was to present a general over of the needs of and support for frail seniors in Italy.

Quantitative data regarding socio-demographic aspects, functional limitations, and the extent of support networks were used to describe the sample, and were obtained via simple univariate/bivariate analyses (calculation of the related percentages), using Microsoft Excel 2024 (Microsoft Corporation, Washington, DC, USA). The physical limitations were graded as follows: “mild” when no activity labelled “not able” was reported; “moderate” when one-two such activities were reported; and “high” and “very high” when three–four and five or more activities were indicated, respectively [43].

The standard phases of the ‘Framework Analysis Technique’ [44] were used to facilitate a deep qualitative analysis of the responses to open questions. They were as follows: extensive reading of the transcribed narratives, definition of macro-/sub-categories to build thematic charts (matrices with categories in columns and cases in rows), and interpretation of the results according to similarities and differences in the answers [28,45]. A thematic content analysis [46] was performed manually, without applying a software, as is acceptable in the literature [47]. This step was facilitated by following the conceptual framework included in the topic guide/questionnaire. The macro-/sub-categories are described in Table 1.

The first author, M.G.M. (MS, female), performed the quantitative analyses for this study. The qualitative analysis was carried out by M.G.M., S.Q. (MS, female), and M.S. (PhD, male). In particular, M.G.M., S.Q., and M.S. cross-analysed the charts by summarising step by step the results from single sites (urban and rural), from single regions, and then for the Italian context by merging the full dataset. G.L. (PhD, male) supervised the whole process, especially the interpretation of the accounts that emerged and related relationships.

With respect to health needs, e.g., due to chronic illness, which can indicate risk of disability (e.g., arthritis/osteoporosis, heart disease/hypertension, diabetes) [48], seniors mentioned support persons who can be considered their main “reference point” for accessing health services (e.g., for booking medical visits and diagnostic tests, consulting the GP). Health emergency situations encompassed various physical complaints, mainly due to heart/circulatory problems, respiratory crises, and fractures following falls. These can require healthcare and medical services and imply the need for established available help [49]. Aside from health emergencies, other vulnerable situations (poor income, poor housing, abuse and neglect, scarce social participation), were not considered to avoid enlarging and thus defocusing both the context and implications, even though these contexts can have health consequences [50].

Overall mobility is defined as the ability to move (including with the use of aids) within both the built internal (i.e., own home/building) and external environments (neighbourhood) [22,51]. In our study, mobility in the home is specified as general movement in/between rooms in one’s home/domestic–internal built environment [22,41]. Conversely, going up/down stairs and bending to pick up an object from the ground were included in ADL and IADL. They were not included in “moving in the home” because they are intended to describe general difficulty with movement regardless of the external/internal environment. They are indeed considered activities that might be required both inside and outside the home, while going up/down stairs may not be relevant for seniors who do not have stairs in their home [11,41].

Further daily activities in our study were included in ADL, i.e., getting into/out of bed and sitting/rising from a chair, and not included in “moving in the home” (defined above as moving between rooms). Other activities were similarly included in IADL, i.e., shopping and managing finances/going to the bank, and not included in moving outside the home, since they imply both moving and, respectively, the ability to decide/manage a purchase and personal finances. Likewise, taking a medication/drug, was maintained within IADL and not included in health needs (defined above as help with managing some illnesses, i.e., using health services) [11,41].

Support was principally provided by family members/children but also friends and neighbours, operators from home services, PCAs, volunteers, and building administrators. Friends and neighbours were considered together since these types of support often coincide later in life, as reported by our respondents. Financial support from children in some circumstances (e.g., to pay for repairs in the home, the cost of a PCA, and the cost of a private medical visit), was also mentioned. Further situations with financial help from children/other persons (e.g., for purchasing aids and for covering the cost of a DHH) did not emerge in the narratives.

In addition, the participants reported cases of self-help/care and the use of mobility aids (walking stick, crutch, walker, and wheelchair). The use of “walking aids” [52] was often reported for going up/down the stairs (with a walking stick or crutch) and moving in/moving outside the home (with a walker or wheelchair). With regard to the latter, the use of aids in some cases was combined with the help of a person. It is worth specifying that health professionals are not included among the main types of support when exploring health issues, since the focus is on persons who help seniors for contacting/accessing/using overall health services, especially in emergencies.

The inclusion of relevant verbatim quotations facilitated the qualitative analysis of findings [53]. Each quotation was coded with “IT” (Italy) and an interview number (increasing from 1–120). Importantly, the quotations were mostly drawn from female participants’ narratives since the sample contained more women than men (90 vs. 30). Moreover, despite the aim of maintaining the respondents’ original phrasing as much as possible, in some cases, a small changes were necessary to increase the clarity of the excerpts without altering their meaning.

A preliminary quantification of statements (i.e., of qualitative/open responses) provided an overall picture of care options for frail older people. In this regard, a conversion mixed-methods integration strategy was adopted (with a “qualitative to quantitative” approach), since the same findings were proposed both qualitatively and quantitatively. Consistency between these quantitative and qualitative analyses was achieved by using the former as a numeric introductory synthesis of accounts that emerged from the narratives and to guide the interpretation of the latter. On the other side, the qualitative findings were used to integrate and support/confirm the quantification of the statements, offering a more in-depth understanding of and meaningful insight into the participants’ experiences. Given the qualitative orientation of the study, with no aim to provide statistical value, and that quantitative data were not the primary results, proper statistical evaluation (e.g., standard deviation/SD and significance level/*p* values) was not performed.

#### 2.2.4. The Trustworthiness of Qualitative Data Analysis

To enhance the trustworthiness of our qualitative findings [54], a deep and comprehensive background literature review was conducted and used to build an initial conceptual framework (transferability), in addition to a careful and detailed description of the study protocol, including field notes on data collection, transcriptions of the narratives and analytic process (dependability and confirmability). Several peer de-briefing sessions among qualified researchers and interviewers (forming a multidisciplinary research team), were carried out, with regular dissemination seminars involving relevant stakeholders and experts in the field, to gradually validate the preliminary findings (credibility).

The team included researchers (the authors of this paper) who are senior gerontologists with extensive experience in quantitative/qualitative studies and knowledge of the health–social needs of frail seniors ageing in place. It is noteworthy that inter-coder reliability was ensured, since three authors (M.G.M., S.Q., and M.S.) independently coded a subset of the transcripts to generate initial codes (40 each), compared the findings, and solved discrepancies through discussion to reach consensus on the coding scheme (i.e., how the same data should be coded). The codes were reviewed when necessary, with the help of G.L.

In summary, the above procedures facilitated reliable analysis [55]. This study also followed the Consolidated Criteria for Reporting Qualitative Research (COREQ) [56]. More detailed information on the study design (setting, sample, recruitment, instruments, data collection, and analysis) and the trustworthiness of the qualitative analysis is available in a previous publication [28], on which Section 2, “Materials and Methods”, is partly based.

## 3. Results

### 3.1. Sample Characteristics

The participants were mostly seniors aged 80 years and above, female, with a primary–middle-school education level, widowed, and living alone without a PCA (Table 2). The majority of respondents (75%) also reported an inability to perform least one activity, and the support network was almost extensive, with help provided by three or more persons in 71% of cases. (Table 2).

More information on the sample is available in a previous publication [28].

### 3.2. Available Supports for Different Needs and Circumstances: Counting the Statements

The main difficulties and needs of older people related to ADL and IADL, in addition to overall health, mobility, and repairs in the home. Other needs did emerge, i.e., for home repair and hiring a PCA. Most support in this respect is provided by family, especially adult children, who still represent an important pillar of caregiving, followed by friends and neighbours, home service operators and PCAs. Cases of self-help and the use of mobility aids were also reported (Table 3; Figure S3 in the Supplementary Material, File S1).

With regard to help performing ADL and IADL, family members are the most frequent “providers” (78%), particularly children (60%), followed by friends and neighbours and private home services, e.g., DHH (both 42%). Public home services (e.g., PHC) and PCAs are less involved (36% and 23%, respectively), and in some cases, volunteers and the use of aids (i.e., for going up/down the stairs) provide help (Table 3; Figure S4 in the Supplementary Material, File S1).

Regarding health needs, the interviewees again mentioned relatives (44%), mostly children (28%), in addition to PCAs (19%) and PHC operators/workers (14%). Friends and neighbours, DHHs, and especially volunteers were mentioned less often. In eight cases, children also helped financially by paying for private medical visits (Table 3; Figure S5 in the Supplementary Material, File S1).

For health emergencies, in most situations, the initial help comes from the family (43%), particularly children (32%), followed by friends and neighbours (18%) and a in few cases, by PCAs and operators from public home services. In some cases (14%), the seniors independently faced the emergency and called for public health assistance (e.g., an ambulance) (Table 3; Figure S6 in the Supplementary Material, File S1).

For mobility in the home, the most important “help” is the use of mobility aids (33%). Some help is provided by PCAs when present (10%), and very rarely from friends and neighbours, home services workers, and volunteers. The family never is of help in this respect (Table 3; Figure S7 in the Supplementary Material, File S1).

The use of aids is crucial for movement when seniors leave the home for the external environment (40%). Other support is predominantly provided by family (17% overall; 12% children) and less by PCAs (9%), friends and neighbours (7%), volunteers (5%), and home services operators (very rarely) (Table 3; Figure S8 in the Supplementary Material, File S1).

When seniors need help with repairs in the home, in most cases (42%), they independently call a professional technician. With regard to help from others, family is again the main provider (36%), especially children (28%), followed by friends and neighbours (15%) and building administrators (12%). PCAs were of help in a few cases. In six cases, the children also helped financially to pay for some home repairs (Table 3; Figure S9 in the Supplementary Material, File S1).

PCAs were often recruited through family members (70%, i.e., 19 out of 27 cases with PCAs), especially children (44%). Friends and neighbours also helped, although less frequently (18%). In four cases, children also helped financially to pay (or integrate) the salary of the PCA (Table 3; Figure S10 in the Supplementary Material, File S1).

### 3.3. Available Supports for Different Needs and Circumstances: Quotations from the Narratives

In this section, several quotations from the qualitative interviews are provided to support and enrich, with more detail, the quantitative data synthetised and presented above. Various types of help (from persons or aids) for different needs are considered, as anticipated in Table 3, Figure S3–S10 (more quotations are available in Supplementary Material, File S2).

#### 3.3.1. Help with Daily Activities

Among children, daughters provide more help with heavy housework or shopping, whereas sons provide more help for technical/bureaucratic/financial issues. Children also help with the administration of drugs.

*My daughter is of help for light cleaning the house and taking medications. (IT_94)*

*My son goes to the bank to withdraw my pension and for paying the bills. (IT_57)*

Brothers and sisters help within the limits of their own age and health.

*My sisters help me sometimes but they are very old and one has heart problems! (IT_94)*

In some cases, nieces (including children of sisters/brothers) and cousins were reported as important sources of help.

*If I need anything, I call my niece on the phone. (IT_64)*

*Two cousins take care of preparing food and cleaning the house. (IT_113)*

However, support from relatives is greatly facilitated by living in close proximity (same city/municipality).

*My daughter lives close to me and helps me every day. (IT_108)*

Friends and neighbours provide help with daily needs and mitigate the feeling of loneliness.

*My friend gives me both material and emotional help! I love her! (IT_97)*

*The neighbour who lives in the apartment above mine is a good friend. I trust her a lot! (IT_36)*

PCAs are hired (mainly for personal care) when seniors become widows and/or have serious health/mobility problems, e.g., following falls. DHHs are hired mainly for housework.

*The PCA was hired when my husband died and I had a heart attack. (IT_80)*

*The PCA is of help for getting in and out of bed. (IT_114)*

*A DHH helps me clean the house, since I cannot do it alone anymore! (IT_115)*

Older people who can hire a PCA or a DHH without problems are in a better economic situation.

*I have two pensions and some annuities thus I can pay for a PCA. (IT_81)*

*I have two pensions. I have enough money to pay a DHH. (IT_83)*

PHCs is sometimes considered insufficient as for number of weekly hours.

*Public assistance is not enough. A PHC cleans the house only a few hours a week! (IT_78)*

Volunteers help with shopping, following payment of a small symbolic fee or as a free service.

*When I need to go shopping I pay five EUR to the volunteers, and they accompany me. (IT_34)*

In some cases, aids, e.g., walking sticks, are essential for managing stairs.

*I need a walking stick and handrail for going up/down the stairs. (IT_84)*

#### 3.3.2. Help with Health Needs and Health Emergencies

The reference “points” for using health services are largely relatives, and (in a few cases) PCAs, PHCs, DHHs, friends and neighbours, and volunteers, sometimes in combination.

*My daughters are my support if I need help with health needs. (IT_49)*

*A PHC and two friends help me when I do not feel well. (IT_41)*

*My nieces, neighbours, the DHH and volunteers help me meet health needs. (IT_45)*

Some respondents specified that their children also provide financially support for private medical visits.

*Private medical visits cost a lot! My son supports me in covering this expense. (IT_113)*

In the case of health emergencies, children are the first available help. However, in these circumstances, the ability to call for help using a mobile phone was crucial.

*When I fell at home, fortunately I was able to call my daughter by mobile phone! (IT_99)*

Friends and neighbours were of help, especially in cases of falls in the home. To a lesser extent, PCAs and operators from public home services also intervened immediately.

*A neighbour helped me immediately when I fell. Then he warned my son. (IT_119)*

*When I fell at home the PCA called the family doctor. (IT_103)*

Some seniors even asked for public health assistance autonomously, to avoid disturbing their family and other persons.

*In the night, I felt bad and thus I called the emergency medical service by myself. My son has his own family! (IT_88)*

#### 3.3.3. Help with Mobility in and Outside the Home

For mobility in the home (moving in/between rooms), respondents often mentioned the use of aids (walking stick or crutch). In a few cases, there was help from a PCA and less from other persons. Support from relatives was never mentioned.

*To move, I use a crutch, but I sometimes need the help of the PCA. (IT_120)*

For mobility outside the home, the use of aids was necessary, including in combination with help from a person, e.g., children and, in some cases, PCAs. This asset provides greater safety to seniors. However, going out alone is a problem because of barriers such as poor maintenance of both streets and sidewalks.

*I go out with a stick and my son. I do not go out alone because I am scared I fall. The streets and sidewalks are all broken! (IT_115)*

*I go to the square with the wheelchair and the PCA. In this way I feel safer! (IT_119)*

In some cases, friends and neighbours and volunteers offer support in going out.

*When I need to go out, my friends are of support with their car. (IT_93)*

*I have limited mobility. I go out with the help of a volunteer. (IT_29)*

Furthermore, public transport is not suitable for seniors with functional limitations.

*I have many difficulties taking a bus. I have weak legs and thus I am afraid of falling. (IT_58)*

#### 3.3.4. Help with Repairs in the Home

When the seniors needed home repairs (e.g., broken pipe, roller shutter, and boiler), the most common solution was independently calling specialised technicians/expert workers. A (less frequent) alternative was calling family members, often children, who then contacted the professionals. Friends and neighbours provided small and easy repairs.

*When I need some repairs in the home, I call some specialized technicians. (IT_44)*

*I call my daughters who in turn provide to call professional technicians. (IT_46)*

*A friend of mine does a bit of everything. He is willing to help me with these things. (IT_58)*

Some respondents specified that their children also support them financially for necessary repairs in the home.

*For some expensive repairs in the home I need financial help from children. (IT_49)*

For these matters in some cases, seniors turn to building administrators. They “know who to call” in every circumstance and have the respective “right” phone numbers.

*I call the building administrator, and he contacts the technician. (IT_83)*

#### 3.3.5. Help with Recruitment of PCA

Seniors reported that the PCA was usually recruited by the family, often children. In some cases, friends and neighbours were also of help in this respect. In all circumstances, however, good references for the PCA were important. Additionally, financial support from children was sometimes necessary to pay/integrate the salary of a PCA.

*The PCA was hired by my son, who also helps me to pay this service. (IT_67)*

*The PCA is well known in the city where I live. She was found by my daughter. (IT_77)*

*My current PCA was found by my friends, who knew her as serious assistant. (IT_60)*

## 4. Discussion

### 4.1. Care Options in Different Circumstances

This study was conducted to explore the available support and care options for frail older people living alone in Italy, with regard to different difficulties and needs/circumstances in daily living. The study is primarily qualitative/descriptive, with a limited and non-representative sample, thus allowing general reflections and discussion on the topic. Despite this, interestingly, the findings indicate that most help is provided by relatives, friends/neighbours, and home services, especially regarding ADL/IADL, health, and mobility, in addition to further specific needs (e.g., for repairs in the home and for hiring a PCA) and cases of self-sufficiency and the use of mobility aids. In Section 4.2 below, the findings are discussed in greater detail by presenting in sequence the main types of available help, with insights on the relevant needs of seniors.

### 4.2. The Family…Is the Family!

In our study, the family provided the most support overall, especially adult children, assisting with all the needs reported by participants, apart from mobility in the home. This is the only need not covered by relatives, probably since our respondents are seniors living alone without cohabiting family members. OECD data [10] confirm our results and indicate how family members play a key role in supporting older people with several daily tasks and in managing their own health/chronic conditions and functional limitations. Other authors found that family members assure material/physical and even psychological support to older people with functional limitations [28,57], especially when public home care and residential services represent are not widely available [58].

With regard to children, our respondents reported that their daughters help more with domestic tasks or shopping, and their sons help with issues related to technical, bureaucratic, and financial matters. In this respect, Albertini and Mantovani [59] also suggest a gendered division of family caregiving to older parents in Italy, with daughters being generally more involved in personal care than sons. Other authors support this and indicate that in Southern European countries, there is a strong belief that children, especially daughters, have the primary/legal responsibility of taking care of their frail older parents; this is also linked to the low availability of public services [60]. Conversely, in northern Europe, support from the family is not considered a “moral obligation”, as public home services are available [61]. Some of our respondents also reported the need for financial support from their children to have private medical visits, which are often prohibitively expensive. According to Guo et al. [62], financial support from relatives is important for the health of seniors, following a decline in their functional/physical abilities, with increasing dependence on support, especially from the family. When health emergencies occur, especially falls in the home resulting in fractures, our seniors again referred to their children as the first available help. Li et al. [63] suggested that children support their older parents in coping with difficult situations when a spouse is absent, as in our study, i.e., regarding seniors who live alone and are, above all, widowed. Children also helped our respondents with mobility outside the home (in the built/external environment). Other authors have stressed the importance of out-of-home mobility for seniors in accessing commodities/facilities/services [64], with environmental barriers representing possible fall-related psychological problems for older persons [65]. Thus, help from children to cope with these difficulties is important, in addition to available public transportation services [66].

Our findings and those of other authors [67] indicate that aside from children, the important “family network” includes other relatives who support frail seniors living alone in Italy, e.g., nieces/nephews, siblings (i.e., brothers and sisters), and cousins. As stressed by Deindi and Brandt [68], seniors with children who are scarcely available or unavailable, including “childless seniors”, often suffer from a crucial care gap when they need help and are no longer able to carry out daily activities independently. In these cases, other significant/close relatives (nieces/nephews and siblings) provide support [69]. ISTAT data [70] suggest that in Italy, among the relatives who help, 39% are cousins and 13% are nieces. Siblings were reported to help in our study, but these individuals are often old themselves, with their own health problems and limited functionalities, so support from them is limited. Some authors [67] indicate that in Italy, siblings provide mostly companionship and psychological support to older relatives, and grandparents have a better health status when they can have at least weekly visits from siblings. ISTAT [70], however, reported that the frequency of contact between non-cohabiting siblings decreases with ageing.

It is worth highlighting that the living/geographical proximity of our older respondents to their relatives (same city/municipality) facilitates support from the latter. According to ISTAT data [71], in Italy, 62% of older people live in the same municipality as their children: 21% live with their children, 15% live in the same building, and 26% live within one kilometre. However, it is important to consider that in recent years, some demographic and social changes (e.g., the decline in births, the greater female employment rate, reduced cohabitation of parents and children) have resulted in a gradual decrease in the number of relatives who can effectively help their own older parents [28]. Additionally, the reduced availability of unpaid family caregivers, especially when intensive care is required, depends on difficult consequences linked to the burden of caregiving, such as a lower possibility of working full-time, difficulty adapting to caregiving work, worse health, and reduced social participation/connectedness [24,72]. Therefore, the rapid ageing of the population in Italy increases the need for care, but the main care resource, family caregivers, is constantly decreasing [73]. Both aspects highlight the need for complementary help beyond the family, in the form of an integrated network. In this respect, seniors mentioned help from friends and neighbours, private and public home services, PCAs, and volunteers, as well as help in the form of mobility aids and self-care, as discussed in depth below.

### 4.3. Friends and Neighbours Are Important

According to some research [72], 60% of frail older people receive support from relatives, friends, and neighbours across OECD countries. According to DOMINA [24], in Italy, the value of hours of LTC provided by relatives, friends, and neighbours, is estimated to be around 2.5% of the EU-27 gross domestic product (GDP), which is higher than public spending on LTC. According to our results for the Italian context, support from friends and neighbours is second to the main help provided by relatives, and it is crucial for almost all the circumstances that we examined, especially for performing ADL and IADL and in case of health emergencies, particularly falls in the home. In the case of falls, the sudden event was immediately “managed” thanks to the support of friends and neighbours, even though the adult children of the seniors were involved and informed of the incident. Other authors [69] stress this aspect, particularly for childless older people with health problems, who are likely to be assisted by extra-familial networks. Seifert and König [74] also indicated that neighbours support older adults when they are in poor health, widowed, and childless.

Importantly, our findings suggest that friends and neighbours are perceived to help mitigate feelings of loneliness among seniors. Other authors [75] found that such relationships involve intimacy and personal matters, with the potential to counteract social isolation. However, the possibility of counting on friends and neighbours follows a natural decreasing trend with age, due to the death of senior friends and historical neighbours or following the relocation of the latter elsewhere, which also implies a lack of confident persons [76]. In this respect, it is worthy to highlight that, as in our results and reported in the literature [28], support from friends and neighbours often coincides in later life. This calls into question once more (as with help from relatives) the importance of housing proximity, with bonds built and consolidated over several years. Bražinová and Chytil [77] also reported that geographic closeness facilitates mutual social support, particularly in difficult situations (e.g., health emergencies), in addition to improving the quality of relationships.

### 4.4. Private and Public Home Services: The Former Have a Cost; The Latter Are Insufficient

Our respondents reported that private home services (e.g., DHH) and public home services (e.g., PHC) primarily support ADL/IADL (mostly by the private sector) and health needs (mainly by the public sector).

DHHs help with housework; in this respect, a good financial situation is a “facilitating” condition for hiring them, even though participants did not explicitly report a need for financial help from relatives/children to pay the salaries of these workers. However, according to DOMINA [24], only 55% of seniors can afford a DHH for five hours a week (about 2400 EUR a year). Our respondents also considered support from PHCs inadequate. Indeed, in Italy, public home-based in-kind services are scarce. ISTAT data [78] highlight that in 2021, SAD was provided to 0.9% of seniors, in addition to the integrated home care service that provides mixed health/social support (Assistenza Domiciliare Integrata in Italian—ADI), which supported only 0.6% of them. Moreover, as highlighted by our respondents, the hours of assistance granted per week are not sufficient. Public home care in Italy is, indeed, essentially provided in the form of weekday and daytime services, which are delivered for a few hours annually, i.e., with a national average of 98 h for SAD and 18 for ADI per user, amounting to an average of about two hours per week of care [73].

Interestingly, as reported by some authors [79], public home services supporting seniors can replace family caregivers in some cases, leading to the so-called crowding-out effect. However, this is more common in northern European countries, which offer robust public services that can effectively relieve families of the caregiving burden. In Italy this circumstance seems more related to the strict eligibility criteria for accessing public services, rather than an inadequate supply of services. These eligibility criteria include the social frailty of users in addition to their functional/physical frailty, such as the absence of a caring family network (e.g., families with children in difficult situations), loneliness/isolation, and poor economic conditions [79]. Additionally, in almost all Italian regions, public home services require co-payment from the user based on specific income brackets; below a certain income, the service is free [73].

### 4.5. Not Everybody’s Budget Has Room for PCA Fees

PCAs largely support our participants for ADL/IADL, health needs, and mobility (in/outside the home). When seniors have serious functional limitations, e.g., following a fall and related possible fractures, or become widows, hiring a PCA is almost necessary. Several authors indicate that the employment of PCAs represents a solution to allow ageing in place, in light of the increasing number of frail seniors needing support, the insufficiency of LTC services, and decreasing availability of family carers [80].

Importantly, seniors who can hire a PCA have better financial situations; in this regard, some participants reported a need for financial support from their children to pay the PCA’s salary. DOMINA [24] clearly indicates a generally poor ability of some Italian seniors to afford a PCA, since their main source of income is a pension. The pension is less than 15,000 EUR a year in 57% of cases, whereas hiring a PCA for 54 h a week and cohabitation costs about 17,000 EUR a year. In this respect, other authors suggest that the need to keep the cost of a PCA low “forces” families to employ irregular/undeclared PCAs, even hiring them outside the formal/regular labour market [80]. It is worth highlighting that in Italy, valid support in these circumstances is available from the IA (EUR 542 per month in 2025) [81]. This allowance, which does not depend on income, is available to persons who are recognised/certified as totally dependent and is often used to hire a PCA for older people [28]. Recent data for Italy in the period 2023–2024 [27] indicate that approximately one in four older people with disabilities receives financial assistance for this condition (e.g., IA).

Support from a PCA can thus be of great help in mitigating the caregiving burden for family. It is, however, important to consider that some authors suggest that PCA care in Italy is mostly supplementary rather than a substitute for help from family members, with a general division of roles between them (personal care/hygiene assistance provided by the former, and financial management provided by the latter), which leads to the so-called crowding-in effect [79]. In such circumstances, relatives are also present to monitor, to some extent, the work of the PCA and to manage the employment relationship with this private assistant [28].

### 4.6. Volunteers and Building Administrators: Further Possibilities

In our study, volunteers support the seniors with ADL/IADL (to go shopping), mobility outside the home (accompaniment for walking), and health needs in some cases (to have medical visits). These services are an important opportunity to bridge the gap (often free of charge), although occasional, when other supports (e.g., from family, friends and neighbours) are not available. The literature also highlights volunteering associations as an important part of informal care, in addition to family and public/private services [28]. Interestingly, ISTAT data [82] indicate that support networks in Italy are composed primarily of the closest family members, i.e., the affective network, and then of the elected ones, i.e., other relatives, friends and neighbours, services, and voluntary associations, especially when seniors feel that these supports can effectively meet their needs. Other data [27] confirm that the informal support network includes volunteering, although for only a small percentage (2%).

With regard to home repairs, our findings highlight that in addition to self-sufficiency and help from relatives and friends/neighbours, seniors sometimes ask for help from their building administrator, as indicated above (Section 3). Indeed, some studies highlight that ageing in place implies a need to manage house repair and maintenance, which can result in high costs, stress, and reduced well-being for seniors. This context can prompt them to move from their homes into residential facilities, or from single/independent dwellings into apartments in a condominium, where dedicated administrators can provide these services and reduce the burden linked to housing upkeep and conservation [83].

### 4.7. Mobility Aids Are Necessary

In addition to the support network described above, mobility aids are crucial for seniors. Our sample indeed includes older people with intermediate mobility, i.e., those with limited mobility both in and outside the home. For the latter, the use of aids is often combined with support from a person, mostly a relative/children, especially when streets and sidewalks are bumpy and uncomfortable. In a few cases, there is help from a PCA.

Previous similar findings [51] suggest that almost 62% of seniors aged 65 years and over in Italy are still able to go out when supported by a person and/or aids (walkers or wheelchairs). ISTAT data [11] also reported that 65% of Italians aged 75 years and over with functional limitations need support from persons or walking aids for moving. The same source reported that 44% of seniors with serious reductions in autonomy aged 65 years and over, and 52% of those aged 85 years and over, complain of a lack of both assistance and aids, especially because they cannot access the latter and/or find the aids they have insufficient or inadequate. Almost half of the seniors living alone have this need, which is critical for those with low income levels (77% vs. 62% with greater economic resources). This latter aspect, however, did not emerge from our findings. Resnik et al. [84] also mentioned the use of walking sticks, walkers, and wheelchairs by seniors. They found walking sticks were the most commonly used aids among people aged over 65 years (72%), whereas walkers and wheelchairs were used less frequently (16% and 7%, respectively). Differing from our results, these authors noted that, in some cases, seniors do not want walking aids due to their perception of social stigma, with a feeling of reduced ability linked to using them, even though mobility aids are recognised as important for enhancing individual independence. Other authors stressed the importance of adopting a sensitive approach to designing assistive devices, since this affects their acceptability by seniors [52].

### 4.8. Self-Sufficiency and Remaining Autonomous

Our findings suggest that, in some cases, seniors try to satisfy their needs by themselves, especially in the case of repairs in the home and for some health emergencies. A previous study [51] reported a similar finding, showing that many older people in Italy are determined to live independently and to find a “way” to overcome difficulties, despite having functional limitations. Self-help is the proactive ability of seniors to manage some challenges, especially when they urgently need help with health emergencies. Similarly, other authors indicate that frail older people even “invent” some strategies for addressing their own daily or emergency needs, thus enhancing their residual abilities and skills [85].

However, in our study, seniors who “helped themselves” also reported an unwillingness to disturb their family or other persons, especially in the case of health needs. Other authors [14] highlighted the key role of children in supporting seniors with chronic diseases and functional limitations, even though in some cases, the seniors themselves were resistant to burdening their children. Other studies suggest that the need for autonomy from family and possible feelings of guilt, especially towards children, lead seniors to avoid enlisting their help [86,87]. According to Canvin et al. [88], seniors adopt self-management techniques to cope with both necessary adaptations to increased functional limitations and low expectations for help. Even when they enlist ‘family first’ for help, they often switch to self-care options.

### 4.9. Implications and Suggestions for Possible Practical Interventions

Frail older people require several social and health-related supports, predominantly to perform ADL and IADL, but also in taking care of their own health and moving in/outside the home. In this respect, available care options and effective interventions are crucial for their well-being and potential to age in place.

In our study, the family emerged as the main source of support, even financially. Policies and practices should, therefore, aim to improve both the well-being of seniors and to support family caregivers by offering them more care allowances, respite care, flexible work (e.g., smart-telework), care leave, and training [10]. Also, technological innovations in care and assistance have the potential to support both caregivers and care receivers through remote monitoring systems and telemedicine [24]. In particular, accessible/usable assistive/wearable devices, for extending the duration of independent living of seniors in the home environment, could be helpful for preventing health emergencies. With regard to the latter, our results indicate the importance of support from friends and neighbours, especially in the case of falls in the home. This suggests a need to strengthen care in the community, through good neighbour initiatives, practical assistance, and social activities, including fall-prevention and surveillance campaigns.

The participants reported receiving help from public home care services; however, this support is considered inadequate, especially for seniors living alone. In Italy, the SAD should indeed be more responsive to the needs of older users and extend beyond the provision of services (personal hygiene, dressing, and house cleaning) for a limited duration and concentrated at specific times of the day, to offer additional support, for example, on holidays and at other times (e.g., evenings/nights) [73]. Given that public home care is currently marginal, in terms of the coverage and intensity of services, and that private paid assistance is expensive, a more integrated formal–informal and public–private mix of services, including PCAs, could work better. This could ensure continuity of care, even at night and during holidays, including emotional/psychological assistance [89]. It is worth mentioning that in Italy, the recent National Law n. 33/2023 (and its Implementing Decree n. 29/2024) aims to promote guidelines, particularly for home services, to promote better social health and formal–informal integration [90].

With regard to PCAs, who support 27 seniors in our study, the main barrier is the high cost, with financial support from children required in some cases. In the light of this, a more effective incentive system, e.g., providing dedicated allowances for hiring PCAs through the regular market, could be implemented [79] to prevent the employment of unregistered, though less expensive, PCAs.

Overall, both seniors and their family caregivers need to be supported with adequate information, e.g., for accessing and using available support services, to obtain walking aids, to adapt housing, and to reduce barriers in the home. In this respect, GPs and other primary care providers can assume a key role for more adequate and effective prevention and treatment and to improve the safety of frail older people [10].

### 4.10. Limitations

This study has some limitations to acknowledge. Its findings are not representative due to the small purposive sample and the inclusion of only three Italian regions, even though they represent three different levels of socio-economic development in the country. This could generate a bias and hamper the (typological) generalisability of the findings to a broader population; an expanded sample could help to address the issue. A simplified definition of frail seniors is applied, i.e., those aged 65 years and over, with functional limitations, living alone, and needing support for ADL and IADL. In this regard, a more holistic approach including more domains, even though complex to manage, could have provided better results. Only older respondents without cognitive impairments were recruited to include individuals who were able to manage the interview autonomously. In this respect, their cognitive status was not assessed by means of a dedicated test, although it was confirmed by the recruiters (based on their own usual evaluations), by the interviewers, and by the relatives of the older persons interviewed. In this study functional limitations were assessed by using a proxy for the seniors’ objective functional status, since this factor was self-reported and not diagnosed [91]. Thus, the respondents’ subjective perceptions could be both over- and underestimated, though they remain important to consider. The focus on health emergencies that occurred in the last three years before the interview could produce biases, since seniors tend to remember and report recent circumstances more often than past events [92]. In this regard, retrospective data remain useful, especially when the accidents have lasting consequences (e.g., falls). Importantly, vulnerable situations that could have additional consequences (e.g., poor income and scarce social participation), aside from explicit health emergencies, were not considered to avoid enlarging the scope the study. For the same reason, this study does not present an in-depth analysis of territorial realities, e.g., regional and urban/rural, to keep the focus on an overall picture of the topic in Italy. Moreover, due to the main qualitative orientation of the study, more in-depth quantitative analyses of the findings and related statistical appraisals were not conducted, even though this could have facilitated further and more accurate conclusions. Finally, absolute values regarding the count of statements in the tables are sometimes very small, suggesting a need for caution when interpreting the related percentage values. It is worth adding that, even though the reproducibility of our study is limited due to the qualitative focus [93], it may offer a basis for more in-depth quantitative investigations on the topic. In this respect, the trustworthiness of the qualitative analysis mentioned above (Section 2.2.4), could indeed represent a strength of our results, especially in light of the collaborative approach and constructive discussions among researchers involved in the study, which were assured in each step even though inter-researcher agreement was not assessed, since we did not measure agreement on the codes assigned to the same data between coders, (e.g., by calculating and reporting a measure of the agreement as the percentage of content units on which coders agree on all the codes applied in the study). Additionally, a preliminary study specifically on the whole study protocol, with detailed information on setting, sampling, and measures, has not been published, which is an additional critical issue to recognise.

## 5. Conclusions

This primarily qualitative/descriptive study allows mostly general conclusions, due to its limited and non-representative sample. However, the findings provide a comprehensive analysis of care options for older Italians with functional limitations in different circumstances and for different needs, mainly for ADL and IADL, health, and mobility. Support is provided primarily by relatives, especially children, but also by proximity networks, home services, and PCAs. Help from public home services is not sufficient, and support from private home services and PCAs is too expensive in some cases. Despite this network, seniors highlight their need for mobility aids and that, in some cases, they independently manage some needs, such as in the case of health emergencies and repairs in the home, calling for necessary help. These findings highlight the central caring role still performed by the family network, which takes on new organizational forms, with the addition of complementary/integrative supports, within overall combined and more articulated care arrangements. This suggests to researchers and policymakers the importance of enhancing the independent living abilities of older people and the need to develop and implement an integrated framework of public–private home services and formal–informal sources of help for them, including financial help.

Future research can conduct in-depth analyses on this topic by considering the following: settings different from the community, e.g., care homes; gender and geographic dimensions, e.g., northern and southern Italy, to take into account territorial differences, especially the decreased provision of home services in the latter; and the needs and types of support available for frail seniors not living alone, e.g., those with old partners or adult children. All these factors, including territorial (e.g., local context, services provided) and relational (e.g., available social networks) dimensions of neighbourhoods, pose possible barriers to and drivers for ageing in place. The various possibilities/combined options for supporting ageing in place, in addition to ageing in care facilities or other housing solutions, should be carefully considered and included in a research agenda aimed at providing adequate spaces for older people.

## Figures and Tables

**Table 1:** Categories for the qualitative analysis.

Macro-Categories: Main Needs	Sub-Categories: Main Types of Support
- ADL ^1^ (e.g., personal care) and IADL ^1^ (e.g., domestic activities) - Health needs (need for a reference person for managing some illnesses, i.e., to use health services, e.g., GP ^2^) - Health emergencies (need for a first available help in case of, e.g., falls, fractures following falls, heart/circulatory problem, respiratory crisis, flu) - Mobility in the home (in the built/internal environment, e.g., in/between rooms of the home) - Mobility outside the home (in the built/external environment, e.g., in the streets) - Repairs in the home (e.g., broken pipe; broken roller shutter, broken boiler) - Recruitment of PCA ^3^	Help from persons - Family (e.g., children, also financially) - Friends and neighbours ^4^ - Operators from private home services (e.g., DHH) ^5^ - Operators from public home services (e.g., PHC) ^6^ - PCA ^3^ - Volunteers - Building administrator
	Self-help (“do-it-yourself”) - To seek for public health help in case of emergency - To call a technician in case of repairs in the home
	Mobility aids: - Walking stick, crutch: for going up/down the stairs and moving in/outside the home; - Walker, wheelchair: for moving in/outside the home

^1^ This includes ADL (basic activities of daily living), IADL (instrumental activities), two specific mobility limitations (going up/down the stairs and bending to pick up an object from the ground), in addition to sensory limitations in hearing and eyesight; ^2^ GP: General Practitioner: ^3^ PCA: Personal/Private Care Assistant; ^4^ Friends and neighbours often coincide in later life; ^5^ DHH: Domestic Home Help; ^6^ PHC: Professional Home Care.

**Table 2:** Sample characteristics (absolute values/n and %).

Characteristics	N = 120	%
Age group (years)		
67–74	17	14
75–79	19	16
80–84	28	23
85 and over	56	47
Gender		
Male	30	25
Female	90	75
Education		
No title	14	11
Primary–Middle school (5 and 3 years)	75	63
High School–University (3–5 years both)	31	26
Level of physical limitations ^1^		
Mild	30	25
Moderate	33	28
High	27	22
Very high	30	25
Marital Status		
Single	16	14
Divorced/separated	16	14
Widowed	88	73
Living Situation		
Alone	93	78
With PCA	27	22
Extent of support network ^2^		
3+	85	71
2	27	22
1	7	6
No help	1	1
Total Cases/Respondents	120	100

^1^ The level of physical/functional limitations is based on 12 ADL-IADL, two specific mobility limitations (going up/down the stairs and bending to pick up an object) and sensory limitations in hearing and seeing. Mild = no activities “not able”, Moderate = 1–2, High = 3–4, Very high = 5 or more; ^2^ Total number of persons who help (relatives, operators from home services, PCAs, friends and neighbours).

**Table 3:** Types of support and different needs (n = number of statements/absolute values) ^1^.

Types of Help ^2^	ADL IADL ^9^		Health Needs ^10^		Health Emergencies (First Help) ^11^		Mobility in the Home		Mobility Outside the Home		Repairs in the Home		Recruitment of PCA ^12^	
	**n**	**%**	**n**	**%**	**n**	**%**	**n**	**%**	**n**	**%**	**n**	**%**	**n**	**%**
Family	94	78	53	44	52	43	-	-	20	17	43	36	19	70
*Children* ^3^	*71*	*60*	*33*	*28*	*38*	*32*	*-*	*-*	*14*	*12*	*33*	*28*	*12*	*44*
Friends and neighbours	50	42	10	8	21	18	1	1	8	7	18	15	5	18
Private services (e.g., *DHH*) ^4^	50	42	10	8	-	-	1	1	3	2	-	-	-	-
Public services (e.g., *PHC*) ^5^	43	36	17	14	3	2	3	2	3	2	-	-	-	-
PCA ^6^	27	23	23	19	4	3	12	10	11	9	3	2	-	-
Volunteers	10	8	4	3	-	-	1	1	6	5	-	-	-	-
Building administrator	-	-	-	-	-	-	-	-	-	-	14	12	-	-
‘Do-it-yourself’ ^7^	-	-	-	-	17	14	-	-	-	-	50	42	-	-
Mobility aids ^8^	10	8	-	-	-	-	40	33	48	40	-	-	-	-
Total respondents	120	100	120	100	120	100	120	100	120	100	120	100	27	100

^1^ The values in the table concern the number of older persons who reported receiving at least one type of help (one case with family helping = even if with more family members help); ^2^ More types of support are possible; ^3^ Both sons and daughters in some cases. Financial help from children (8 cases to pay private medical visits, 6 cases to pay some repairs in the home, and 4 cases to pay/integrate the salary of the PCA) is not specified in the table, where only general help from children for various needs is reported; ^4^ DHH = Domestic Home Help; ^5^ PHC = Professional Home Care; ^6^ PCA = Personal/Private Care Assistant; ^7^ ‘Do-it-yourself’, i.e., to seek for public health help in case of emergency and to call a technician if home repairs are needed; ^8^ The use of mobility aids was also mentioned for going up/down the stairs (ADL/IADL category, e.g., walking stick), and moving in/outside the home; ^9^ Including ADL, IADL, going up/down the stairs, bending to pick up an object from the ground, and sensory limitations in hearing/eyesight; ^10^ Help from persons who can be considered a “reference point” for these needs; ^11^ Emergencies were physical complaints/illnesses (50 cases), and falls (47 cases). Episodes occurred mainly in the home (86), and less outside (11 falls); ^12^ Percentages are calculated with n = 27, i.e., the number of seniors with a PCA.

## Data Availability

The data presented in this study (i.e., absolute values, relevant quotations) are included in the article and Supplementary Material. Some quantitative data (e.g., socio-demographic) are openly available in Mendeley at https://doi.org/10.17632/3ryrpz224h.2 (accessed on 6 February 2026). The qualitative dataset with verbatim transcriptions in Italian is not publicly available due to ethical restrictions, i.e., it contains sensitive information on the respondents (e.g., names and locations) that could compromise their privacy.

## Supplementary Materials

The following supporting information can be downloaded at https://www.mdpi.com/article/10.3390/healthcare14111432/s1: File S1, Figures S1–S10; File S2, Additional Quotations.

## Abbreviations

The following abbreviations are used in this manuscript:

ADI	Integrated Home Care (Assistenza Domiciliare Integrata)
ADL	Activity of Daily Living
DHH	Domestic Home Help
EU	European Union
EUR	Euro
GDPR	General Data Protection Regulation
GP	General Practitioner
IA	National Disability Attendance Allowance (Indennità di accompagnamento)
IADL	Instrumental Activity of Daily Living
IN-AGE	Inclusive Ageing in Place
LTC	Long-Term Care
PCA	Personal Care Assistant
PHC	Professional Home Care
POLIMI	Polytechnic of Milan
SAD	Home Care Service (Servizio di Assistenza Domiciliare)
