# Supplementary material for: Care Needs and Care Options for Frail Older People Living Alone in Italy: An Exploratory Mixed Study
Source: Healthcare (Basel). 2026 May 22;14(11):1432. doi: 10.3390/healthcare14111432 (PMC13257076; doi:10.3390/healthcare14111432)
Supplement: Supplementary file 1 [file healthcare-14-01432-s001.zip › Supplementary Material File S2 - Additional quotations.pdf]

Supplementary Material File S2 – Additional quotations

Help with Daily Activities

I go shopping always with the help of my daughter. (IT_20)
My brother comes to help me in some way with the shower. (IT_101)
My cousin is like a sister. She looks after me, comes whenever I need her. (IT_74)
My friend accompanies me to the bank and to go shopping if necessary. (IT_52)
A very kind neighbour does the shopping for me. He is a friend for me. (IT_14)
I fell twice with fracture of the femur. Thus my family hired a PCA to take care of me. (IT_60)
I need the help of the PCA to sit on the chair. (IT_30)
The girl from home care service who cleans my house helps me a lot! (IT_50)
I use a crutch to climb the stairs. I need to move very slowly and I have to hold on to the handrail. (IT_120)

Help with Health Needs and Health Emergencies

My sons and the PCA help me if I need to use health services. (IT_51)
When I feel bad I call the DHH who has helped me for many years. (IT_72)
Help with health is provided mainly by a neighbour. She is a good girl, I trust her. (IT_36)
A volunteer helps me when I need to have a medical visit. (IT_43)
When I had a respiratory crisis, I called my children by mobile. (IT_100)
When I fell I called a friend, who lives downstairs, who took me to the hospital. (IT_33)
When I fell, I called the ambulance without involving my children. They live too far away! (IT_14)

Help with Mobility in and Outside the Home

I use a stick and I have a crutch for moving in my home. (IT_112)
Inside the house I use the walker. I need it especially when I sit and then I get up. (IT_53)
I only go out with a stick and when my children take me by a car, I do not move alone. (IT_105)

Help with Repairs in the Home

I call expert workers: the plumber for questions regarding the water, the electrician for questions regarding electricity, and so on. (IT_54)
The PCA gives me all the suggestions and information regarding who I should call. She is extremely collaborative. (IT_79)

Foreign presence PCAs, DHHs, PHCs, friends and neighbours.

PCA comes from Romania. She is a wonderful person! (IT_105)
DHH comes from the Philippines. She is a good woman! (IT_93)
PHC is a Nigerian woman. I have a very good relationship with her. (IT_41)
I have a Slovenian friend who helps me with shopping. (IT_52)
