# Supplementary figures and images for: Care Needs and Care Options for Frail Older People Living Alone in Italy: An Exploratory Mixed Study
Source: Healthcare (Basel). 2026 May 22;14(11):1432. doi: 10.3390/healthcare14111432 (PMC13257076; doi:10.3390/healthcare14111432)

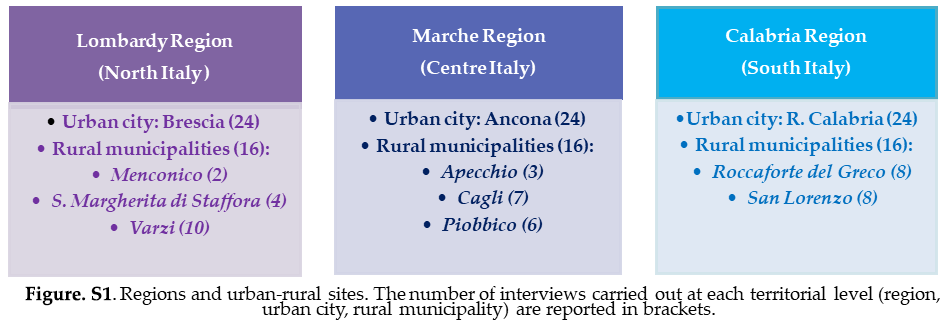

Supplement: Supplementary file 1 [file healthcare-14-01432-s001.zip › Supplementary Material File S1 - Figures 1-10/Figure S1 Sites.TIF]

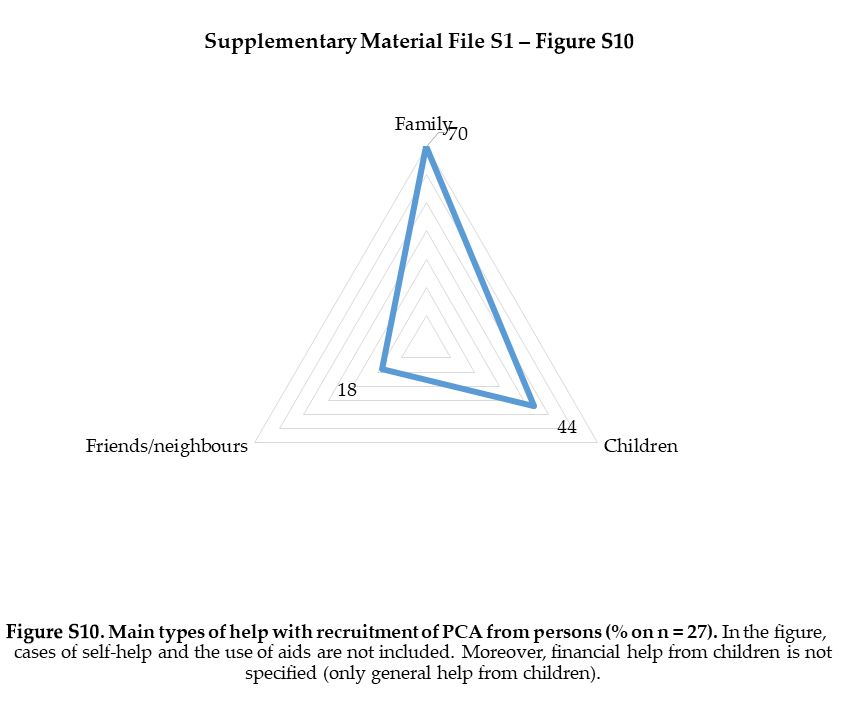

Supplement: Supplementary file 1 [file healthcare-14-01432-s001.zip › Supplementary Material File S1 - Figures 1-10/Figure S10 Help with recruitment of PCA.TIF]

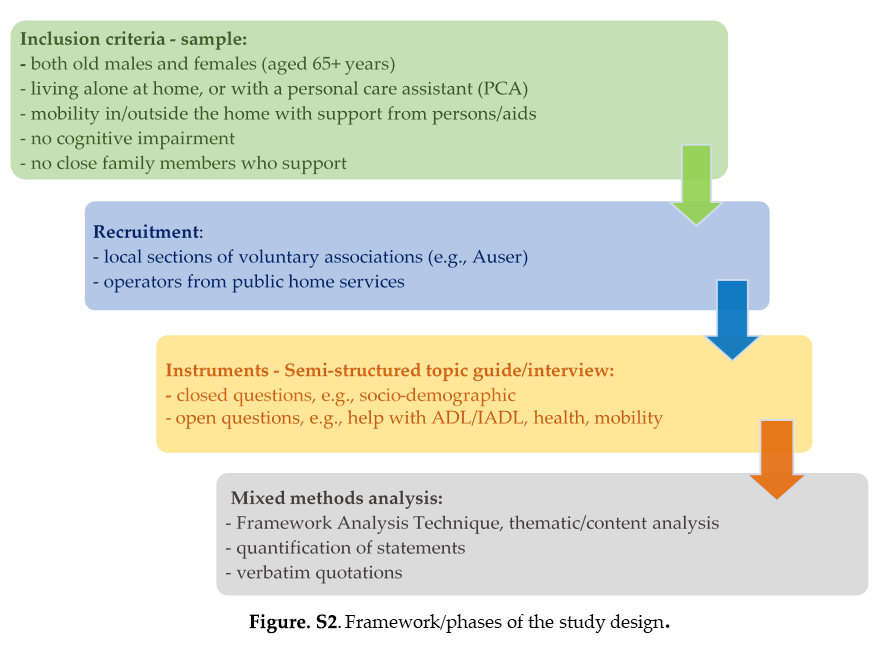

Supplement: Supplementary file 1 [file healthcare-14-01432-s001.zip › Supplementary Material File S1 - Figures 1-10/Figure S2 Study design.TIF]

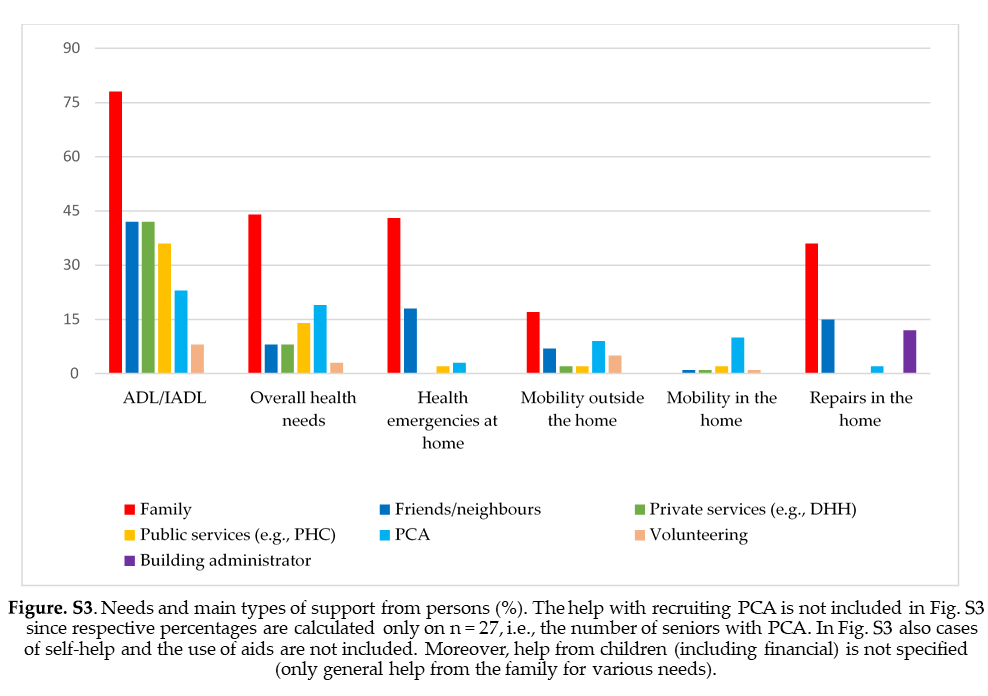

Supplement: Supplementary file 1 [file healthcare-14-01432-s001.zip › Supplementary Material File S1 - Figures 1-10/Figure S3 Needs and supports.TIF]

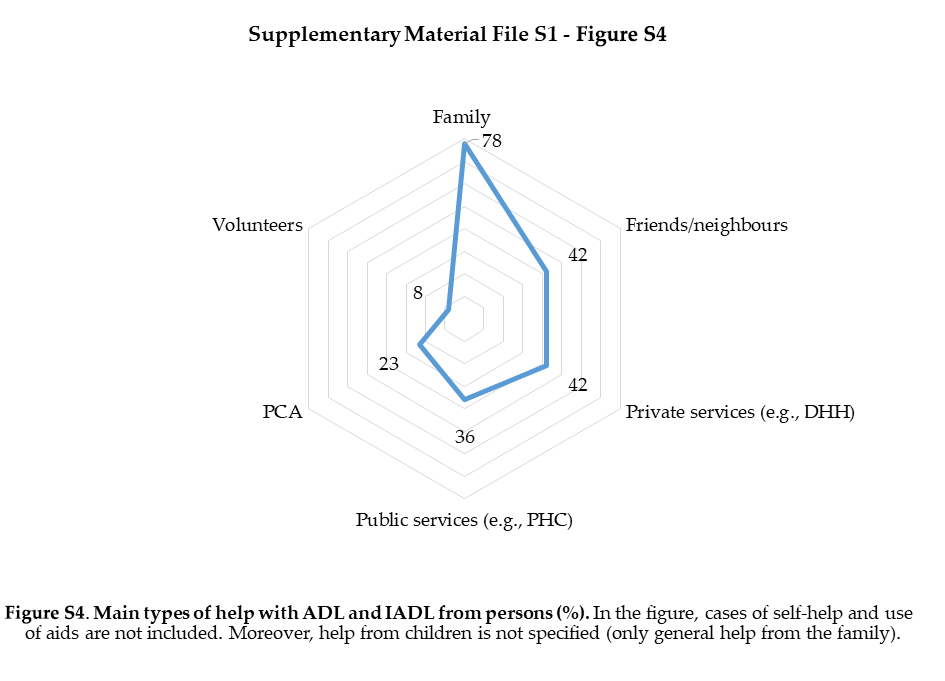

Supplement: Supplementary file 1 [file healthcare-14-01432-s001.zip › Supplementary Material File S1 - Figures 1-10/Figure S4 Help with ADL IADL.TIF]

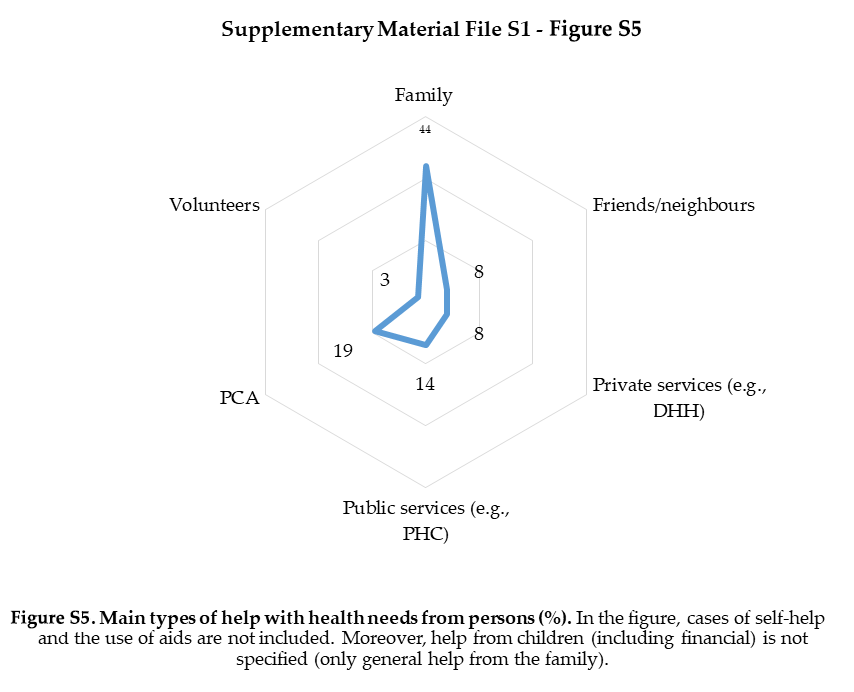

Supplement: Supplementary file 1 [file healthcare-14-01432-s001.zip › Supplementary Material File S1 - Figures 1-10/Figure S5 Help with health needs.TIF]

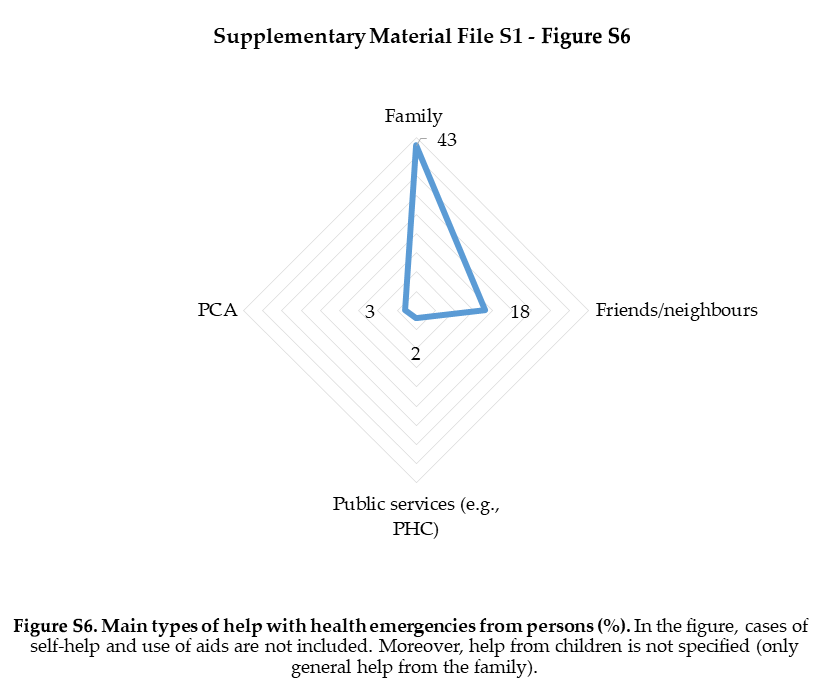

Supplement: Supplementary file 1 [file healthcare-14-01432-s001.zip › Supplementary Material File S1 - Figures 1-10/Figure S6 Help with health emergencies.TIF]

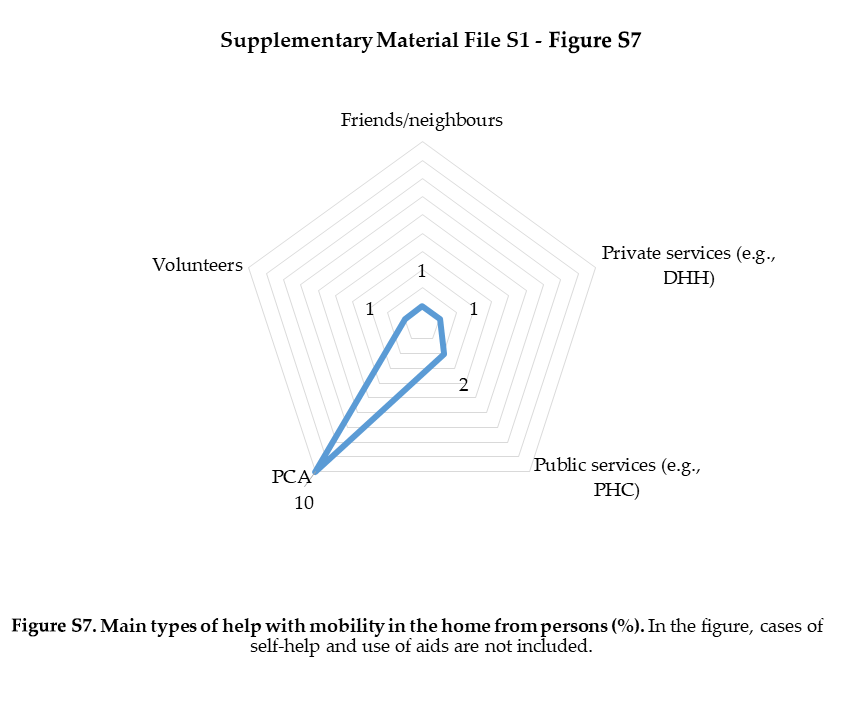

Supplement: Supplementary file 1 [file healthcare-14-01432-s001.zip › Supplementary Material File S1 - Figures 1-10/Figure S7 Help with mobility in the home.TIF]

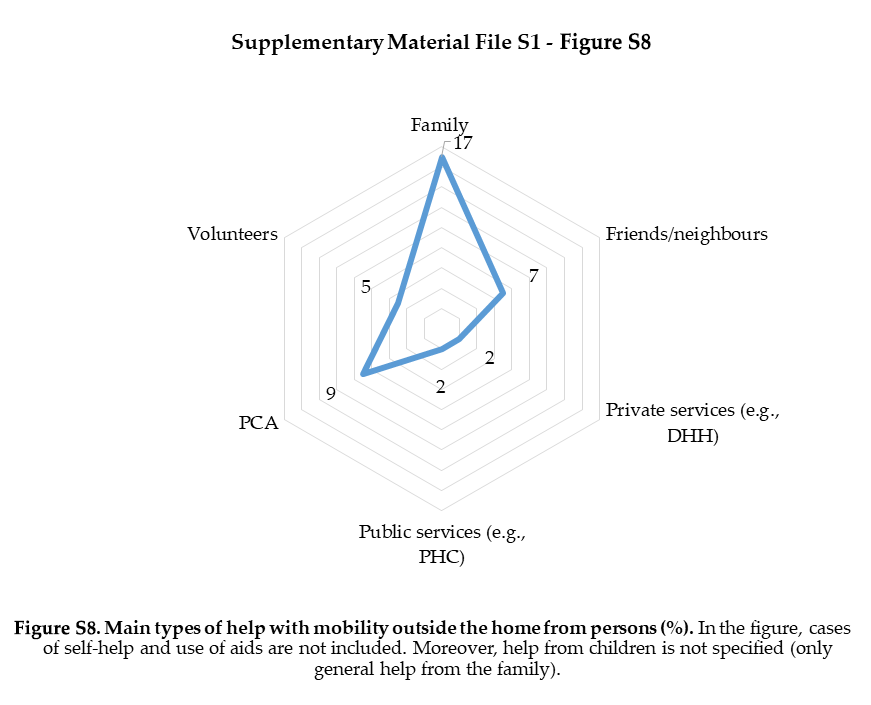

Supplement: Supplementary file 1 [file healthcare-14-01432-s001.zip › Supplementary Material File S1 - Figures 1-10/Figure S8 Help with mobility outside the home.TIF]

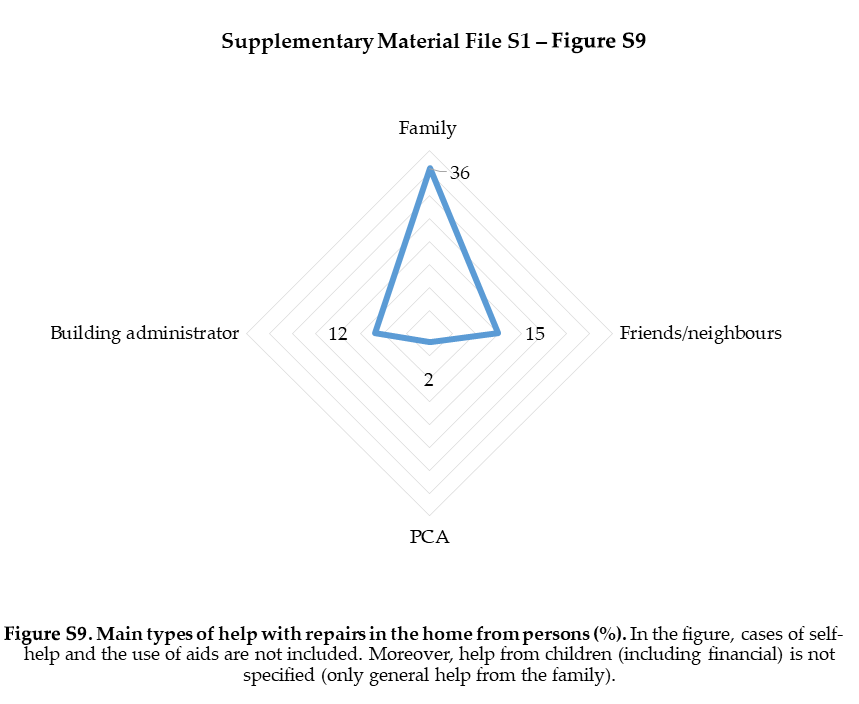

Supplement: Supplementary file 1 [file healthcare-14-01432-s001.zip › Supplementary Material File S1 - Figures 1-10/Figure S9 Help with repairs in the home.TIF]
